# Valorization of Sugarcane Straw for the Development of Sustainable Biopolymer-Based Composites

**DOI:** 10.3390/polym13193335

**Published:** 2021-09-29

**Authors:** Jorge R. Robledo-Ortíz, Alan S. Martín del Campo, Juan A. Blackaller, Martín E. González-López, Aida A. Pérez Fonseca

**Affiliations:** 1 Department of Wood, Cellulose and Paper, University of Guadalajara, Zapopan 45510, Mexico; jorge.robledo@academicos.udg.mx; 2 Department of Chemical Engineering, University of Guadalajara, Guadalajara 44430, Mexico; salvador.martindelcampo@alumnos.udg.mx (A.S.M.d.C.); juan.blackaller@alumnos.udg.mx (J.A.B.); martin.gonzalez@alumnos.udg.mx (M.E.G.-L.)

**Keywords:** biopolymers, sugarcane straw, biocomposites, agro-industrial wastes

## Abstract

Sugarcane straw (SCS) is a common agro-industrial waste that is usually incinerated or discarded in fields after harvesting, increasing the importance of developing added-value applications for this residue. In this study, sustainable biocomposites were produced, and the effect of sugarcane straw as a filler/reinforcement of commercial biopolymers was evaluated. Biocomposites were prepared using polylactic acid (PLA), polyhydroxybutyrate (PHB), polyhydroxybutyrate-co-hydroxyvalerate (PHBV), or green polyethylene (Green-PE) with different fiber contents (20, 30, and 40 wt.%). Dry-blending followed by compression molding was used for the biocomposites preparation. The results showed that PLA, PHB, and PHBV biocomposites retained the same impact strength as the neat matrices, even with 40 wt.% of sugarcane straw. The flexural and tensile modulus of PLA, PHB, and PHBV biocomposites increased with 20% of SCS, whereas, in Green-PE biocomposites, these properties increased at all fiber contents. Since any compatibilizer was used, both the flexural and tensile strength decreased with the addition of SCS. However, even with the highest content of SCS, the tensile and flexural strength values were around 20 MPa, making these materials competitive for specific industrial applications.

## 1. Introduction

Lignocellulosic materials are commonly found in ecosystems. In fact, they are the most abundant biopolymer on earth [1]. The global annual production of lignocellulosic biomass is estimated at around 181.5 billion tons [2]. Agriculture wastes are an important type of lignocellulosic biomass, such as bagasse, palm residues, corncob, and straw, among several others [1,3,4]. These lignocellulosic materials have been widely used to produce composites due to their advantages of possessing biodegradability, a low cost, a lower density, and less abrasiveness than synthetic fibers [5].

**Figure 1:**
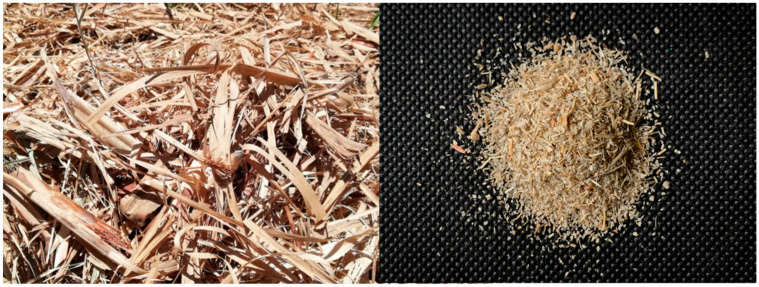
Sugarcane straw.

The sugarcane straw (SCS, Figure 1) is a waste generated from the sugar industry. The stem and the leaves are cut together during the sugarcane harvest, and a cleaning system separates the cane from the straw. The stem is collected for sugar production, while the remaining straw is discarded on the ground. The SCS comprises (dry basis) 62% of dry leaves, 32% of green leaves, and 7% of tops [6]. The average chemical composition of SCS is 37% of glucans, 28% of hemicellulose, 24% of lignin, and 5% of ash [7], which is similar to the sugarcane bagasse composition (41%, 27%, 20%, and 3%, respectively).

**Figure 2:**
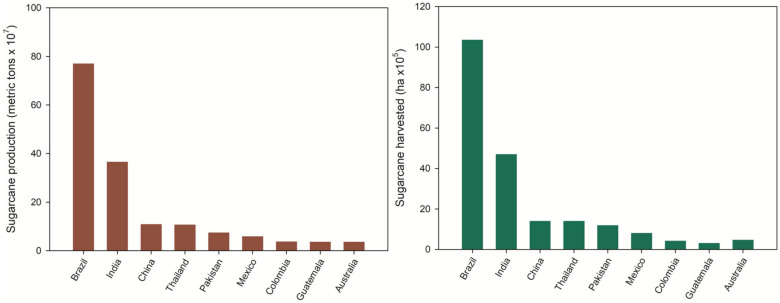
Global sugarcane production.

According to the Nation Master database, Mexico is the sixth global producer of sugarcane, with around 57,000,000 metric tons per year, representing 791,762 ha of harvested fields (Figure 2). The remaining straw represents nearly 28% of the total production [8]. Brazil is the primary producer of sugarcane globally, producing 140 kg of SCS per ton of cultivated sugarcane [9]. Despite the similarity in the chemical composition of both wastes, most research focuses on bagasse valorization, leaving aside the SCS. A possible explanation is that, while bagasse is principally generated by the sugar industry, where a significant interest in obtaining profits from the process exists, the SCS remains with the harvesters, where it is mainly used as fuel for mill boilers, reincorporated into the cycle as a nutrient for soil, or used as cattle food [9,10]. Such actions cause the environmental concern of governments and the sugarcane harvesters, making it necessary to explore new options to exploit this agro-industrial waste [11]. The high abundance of SCS represents an economic opportunity to develop added-value products.

Different applications of SCS have been reported in the literature. For instance, Moraes et al. [12] analyzed the products of SCS pyrolysis as an alternative to the typical burning process, producing bio-oil, bio-gas, ashes, and charcoal, adding value to this material. SCS can be used to produce energy; regarding this topic, Brazil is a world leader in renewable electricity generation [11]. In addition, SCS has been tested as a silica source [13]. Cherubin et al. [14] evaluated the use of different amounts of SCS as a nutrient for soil, concluding that the partial removal of the straw positively affects fertilizer consumption. Other studies have evaluated the obtention of cellulose from SCS and have developed textile fibers with a high tenacity [9]. Frías et al. [15] evaluated the SCS as a pozzolanic material, showing a very high pozzolanic activity. Bilatto et al. [16] performed an organosolv pretreatment of SCS to produce a cellulose nanocrystal and found a significant increase in the crystallinity, opening the possibility for other applications for this waste.

Currently, in the biocomposites field, a great interest in the utilization of sugarcane bagasse exists, which has been successfully used as reinforcement for polymer matrices. Luz et al. [17] obtained cellulose from sugarcane bagasse and prepared composites with polypropylene. They observed that adding 20 wt.% of cellulose slightly decreased the tensile strength while the flexural strength increased. Mulinari et al. [18] modified sugarcane bagasse with zirconium oxychloride, enhancing the mechanical properties of high-density polyethylene composites. Cerqueira et al. [19] pretreated sugarcane bagasse with a sulfuric acid solution followed by delignification, observing increases in polypropylene’s tensile and flexural properties. Even though SCS possesses similar features compared to sugarcane bagasse, such as its chemical composition, high content of cellulose and lignin, and the advantage of not having residual sugars, its use to prepare polymer biocomposites has not yet been adequately explored.

The current concern for the use of eco-friendly materials has caused an increasing demand for developing biopolymers. Among these materials, the thermoplastic starch (TPS) is based on renewable plants [20], the polylactic acid (PLA) and green polyethylene (Green-PE) are synthesized from renewable sources [21,22,23], and the polyhydroxyalkanoates are intracellularly synthesized from microbial fermentation, e.g., polyhydroxybutyrate (PHB) and its copolymer polyhydroxybutyrate-co-hydroxyvalerate (PHBV) [5]. Besides, these biopolymers can be produced from different renewable sources, and the PLA, PHB, and PHBV are biodegradable materials. However, the replacement of conventional oil-based polymers is complicated due to the significant difference in costs. Researchers have recently looked towards alternatives to using agro-industrial wastes as reinforcements or fillers for these biopolymers, showing interesting results. For instance, Kuciel et al. [24] prepared Green-PE composites with wood particles using extrusion followed by injection molding. The addition of wood particles resulted in a higher tensile modulus and a loss of tensile strength. Wang et al. [21] evaluated the effect of sugarcane bagasse on PLA biocomposite properties. The tensile and flexural properties of PLA decreased with the addition of sugarcane bagasse, even with coupling agents, which was similar to the results reported by Bartos et al. [25]. Da Silva Pinto et al. [26] incorporated sugarcane fibers to PHB and reported a decreased brittleness. Ehman et al. [27] produced Green-PE/sugarcane bagasse 3D-printed biocomposites using maleic anhydride as a compatibilizer. They reported improvements in the tensile strength and modulus.

As mentioned, several research papers about sugarcane bagasse composites have been published lately, and, despite the similarity in the chemical composition of SCS with the sugarcane bagasse, there is a lack of information regarding sugarcane straw biopolymer composites. Hence, this research focuses on evaluating sugarcane straw (SCS) as a feasible reinforcement/filler for different biopolymers (PLA, PHB, PHBV, and Green-PE), aiming to both reduce the final cost of the potential products while maintaining competitive mechanical properties, and widen the possible applications of these materials. For this purpose, a simple method was used to process these materials, consisting of dry-blending followed by compression molding, aiming to inspire industry and academic experts to develop added-value products to sugarcane straw waste without increasing processing costs.

## 2. Materials and Methods

### 2.1. Materials

The biopolymers used were PLA Ingeo 3251D from Nature Works LLC (Minnetonka, MN, USA), PHB Y3000 and PHBV Y1000 supplied by Tianan-Enmat (Ningbo, China), and ICO GREEN linear low-density polyethylene (referred to as Green-PE) from A. Shulman (San Luis Potosí, SLP, Mexico). The main physical properties of these biopolymers are shown in Table 1.

Sugarcane straw (SCS) was collected after the mechanical harvesting process at Autlán—El Grullo, Jalisco, Mexico. The SCS was shaken to remove dust and impurities and washed by placing it in containers with water for 24, then separated by decantation and dried outdoors. Afterward, the straw was ground and sieved to obtain particles retained between 50 and 70 mesh.

### 2.2. Biocomposites Preparation

The biocomposites were prepared using 20, 30, and 40 wt.% of SCS, along with PLA, PHB, PHBV, or Green-PE as the biopolymer matrix. Before blending, all of the materials were oven-dried for 24 h at 60 °C. The components were dry-blended in a grinder with dull blades (Hamilton Beach model 80350R, Glen Allen, VA, USA). Afterward, the blends were compression-molded to obtain sheets of 2 mm × 123 mm × 123 mm in a hydraulic press for 10 min at 180 °C and 200 bar. The biocomposite samples were cut with a laser machine Guian Gn640MS (Puebla, PUE, Mexico) into different geometries to perform further characterization.

### 2.3. Physical and Mechanical Characterization

The mechanical properties were tested at room temperature. Charpy impact test was performed according to ASTM D6110 in an Instron Ceast model 9050 (Norwood, MA, USA) impact machine, testing 10 notched samples for each composition. Tensile and flexural properties were determined in a universal machine Instron 3345 (Norwood, MA, USA), testing seven samples per composition. The tensile test was carried out following the ASTM D638 standard using type IV specimens and a crosshead speed of 5 mm/min. The flexural test was performed according to ASTM D790 at a crosshead speed of 2 mm/min. Statistical analysis of variance (ANOVA) and Tukey tests with a 95% confidence level were carried out for the mechanical properties results using MATLAB^®^ software (MathWorks, Natick, MA, USA). In the figures, different letters indicate significant differences (*p* < 0.05) between the SCS contents for each biopolymer matrix.

SEM micrographs of fractured surfaces from the tensile test were acquired in a TESCAN MIRA3 LMU electron microscope (Brno, Czech Republic). The samples were coated with Au for 120 s under vacuum using an SPI Module Sputter Coater (West Chester, PA, USA).

Water absorption tests were conducted according to ASTM D570 using five samples of 60 mm × 13 mm × 2 mm per composition. The samples were dried and weighed before being immersed in distilled water at 55 °C for 500 h. After specific periods, samples were removed from the water, wiped with a dry cloth to remove excess water, and weighed. The amount of absorbed water (*M_t_*) was quantified according to Equation (1):

(1) $$M_{t}\left(\%\right)=\frac{w_{i}-w_{0}}{w_{0}}\times 100$$

where *w_i_* and *w*_0_ are the weight of the sample after immersion and the initial weight, respectively.

Additionally, the diffusion coefficients of the biocomposites were determined using a hindered diffusion model, which assumes that absorption is related to free-volume availability and polymer–water affinity. This model is described by Equation (2) [28]:

(2) $$\frac{M_{t}}{M_{\infty }}=1-\frac{\beta }{\left(\beta +\alpha \right)}e^{-\alpha t}-\frac{\alpha }{\left(\beta +\alpha \right)}\frac{8}{\pi^{2}}e^{\frac{-D\pi^{2}t}{l^{2}}}$$

where *M_t_* is the water absorption at any time, *M_∞_* is the moisture uptake at equilibrium, *D* is the diffusion coefficient, t is the time of test, *l* is the thickness of the samples, and *β* and *α* are dimensionless parameters related to the probability of free molecules to become bound and bound molecules to become free, respectively.

## 3. Results and Discussion

### 3.1. Mechanical Properties

#### 3.1.1. Impact Strength

**Figure 3:**
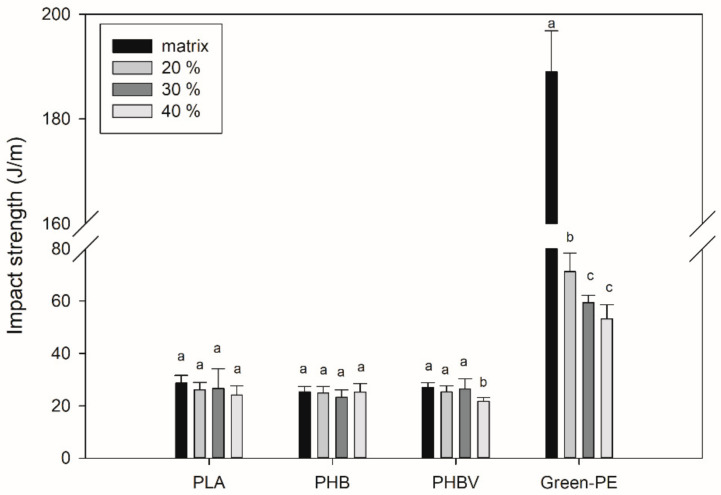
Impact strength of the biocomposites. Different letters (a–c) on the bars indicate significant differences (*p* < 0.05).

Figure 3 shows the impact strength of the biocomposites. The addition of SCS to PLA, PHB, and PHBV did not drastically affect the toughness of the material. For instance, PLA has an impact strength of 28 J/m, which slightly decreased to 26 J/m and 24 J/m with 30 and 40 wt.% of SCS, respectively. For PHB biocomposites, the impact strength remained at around 25 J/m at all fiber contents, which is practically the same value as neat PHB. Similar results were obtained in PHBV biocomposites, retaining an impact strength of 27 J/m with a SCS content of up to 30 wt.% It has been reported that natural fibers do not affect or even increase the impact strength of biopolymers, which is associated with the aspect ratio and surface area of the fibers that promote a better energy absorption during the fracture [29,30,31]:

Conversely, the addition of SCS to Green-PE decreased the impact strength from 190 J/m to values of around 50 J/m. The high elasticity of the Green-PE makes it capable of absorbing a significant amount of energy during the impact test. However, SCS increased the Green-PE stiffness, and, consequently, its impact strength was reduced. A similar behavior was reported for agave and coir fibers used as reinforcement for Green-PE [32] and bamboo fibers in PHB biocomposites [33]. These results are of utmost importance, since they show that it is possible to add SCS to biopolymer matrices and process them by simple techniques to lower the final product cost while still showing a competitive impact strength, which is important for different specific applications. Table 2 shows a comparison of the impact strength of the produced biocomposites with other systems based on the studied matrices, showing that competitive values were obtained.

#### 3.1.2. Tensile Properties

**Figure 4:**
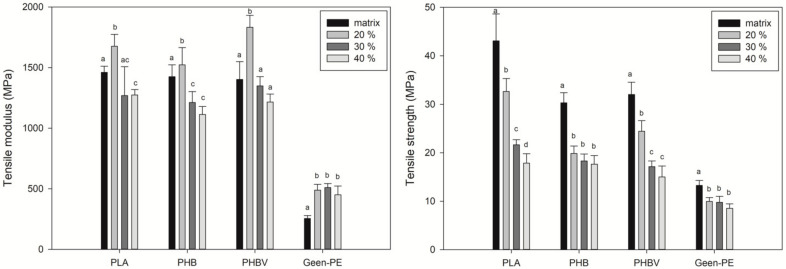
Tensile properties of the biocomposites. Different letters (a–c) on the bars indicate significant differences (*p* < 0.05).

Figure 4 shows the tensile properties of the biocomposites. PLA, PHB, and PHBV are highly stiff polymers due to their high crystallinity [44], and their combination with natural fibers (also known for their high stiffness) results in biocomposites with a higher tensile modulus. PLA, PHB, and PHBV showed a tensile modulus of 1460, 1425, and 1400 MPa, respectively, and the addition of 20 wt.% of SCS improved this property up to 1675, 1523, and 1832 MPa. On the other side, at higher SCS contents, the tensile modulus is around values between 1100–1300 MPa due to the low dispersion and agglomeration at a higher fiber content [45].

Even though the tensile modulus is generally enhanced with natural fiber addition, this behavior usually occurs in composites prepared by twin-screw extrusion or melt-blending, which assure the well mixing and dispersion of the components, achieving higher levels of compaction [30,39,46]. SCS enhanced the Green-PE biocomposite stiffness, showing tensile modulus values that were 100 and 79% higher than the pure matrix, with 30 and 40 wt.% of SCS content. Similar results were reported by Ferrero et al. [47] by adding *Posidonia oceanica* seaweed to Green-PE. It is noteworthy that the dry-blending method significantly reduces costs, since simpler equipment is required compared to melt-blending techniques.

The addition of SCS to all of the biopolymers decreased the tensile strength (Figure 4). The incorporation of natural fibers into hydrophobic polymeric matrices results in a weak interfacial adhesion due to the high hydrophilicity of the fibers [48]. Improving the tensile strength requires a good fiber–matrix interfacial adhesion, as well as other factors, such as high aspect ratio fibers [29,49]. Hence, the lowest tensile strength was observed at 40 wt.% of SCS. This property decreased in the following order: in PLA from 43 to 18 MPa, in PHB from 30 to 17 MPa, in PHBV from 32 to 15 MPa, and in Green-PE from 13 to 8 MPa. In this sense, a higher fiber content causes stress transfer from fiber to fiber, instead of the matrix to the reinforcement, due to agglomeration [50]. Similar decreases in the tensile strength have been reported by several authors when any coupling agents were used for natural fibers, such as agave, wood, bamboo, coconut, jute, and rice straw [51,52,53,54]. In addition, compared to other biocomposites, such as with thermoplastic starch as the matrix, similar values (around 20 MPa) have been reported when using bagasse fiber and Nile rose residues as reinforcements [20,55]. Despite the high losses in this property, it is important to mention that 30 wt.% of SCS could be used as a filler material for biopolymers and could still have acceptable properties for specific applications. Again, it is important to point out that the purpose of this study was not precisely to increase the properties of the used biopolymers but rather to evaluate the possibility of taking advantage of the SCS as a filler in order to develop value-added products.

#### 3.1.3. Flexural Properties

**Figure 5:**
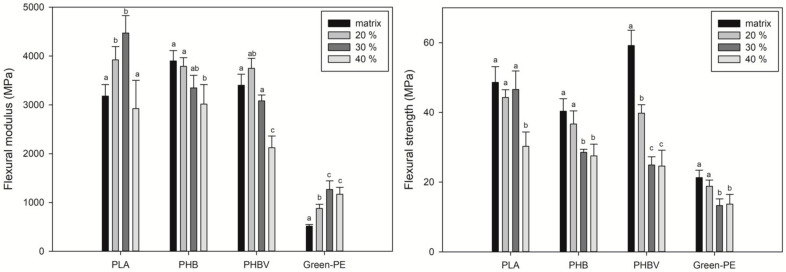
Flexural properties of the biocomposites. Different letters (a–c) on the bars indicate significant differences (*p* < 0.05).

Flexural properties are presented in Figure 5. The PLA flexural modulus increased from 3180 to 4473 MPa with 30 wt.% of SCS. Similarly, Green-PE biocomposites’ flexural modulus significantly increased from 514 MPa to 1267 MPa with 30 wt.% of SCS. On the other hand, the PHB flexural modulus decreased from 3900 to 3400 MPa with 30 wt.% of SCS. In PHBV, a slight increase was obtained with 20 wt.% of SCS from 3400 to 3750 MPa, whereas this property decreased at higher contents. Despite the decrease in the flexural modulus at high SCS contents, especially at 40 wt.%, it remained at acceptable values for all of the studied biopolymers, especially in comparison with commercial high-density polyethylenes [56,57].

The flexural strength results coincide with those observed in the tensile strength, where the incompatibility between SCS and the biopolymers makes the stress transfer ineffective and weakens the material. PLA biocomposites had the best flexural strength results, showing that up to 30 wt.% of SCS could be used without affecting this property. The high stiffness of some natural fibers had been reported to cause a positive effect on the PLA flexural strength [58,59]. Although the flexural strength of PHB, PHBV, and Green-PE biocomposites decreased with the addition of SCS, similar flexural strength values can be obtained at 30 and 40 wt.% of SCS. It is remarkable that, even at 40 wt.% of SCS, PHB and PHBV biocomposites showed competitive values (28 and 24 MPa, respectively) compared with high-density PE and biodegradable aliphatic polyester [60,61]. In Green-PE, 20 wt.% of SCS induced a slight reduction in the tensile strength from 21 to 19 MPa, whereas, at 30–40 wt.% it dropped to 13 MPa.

### 3.2. Morphology

**Figure 6:**
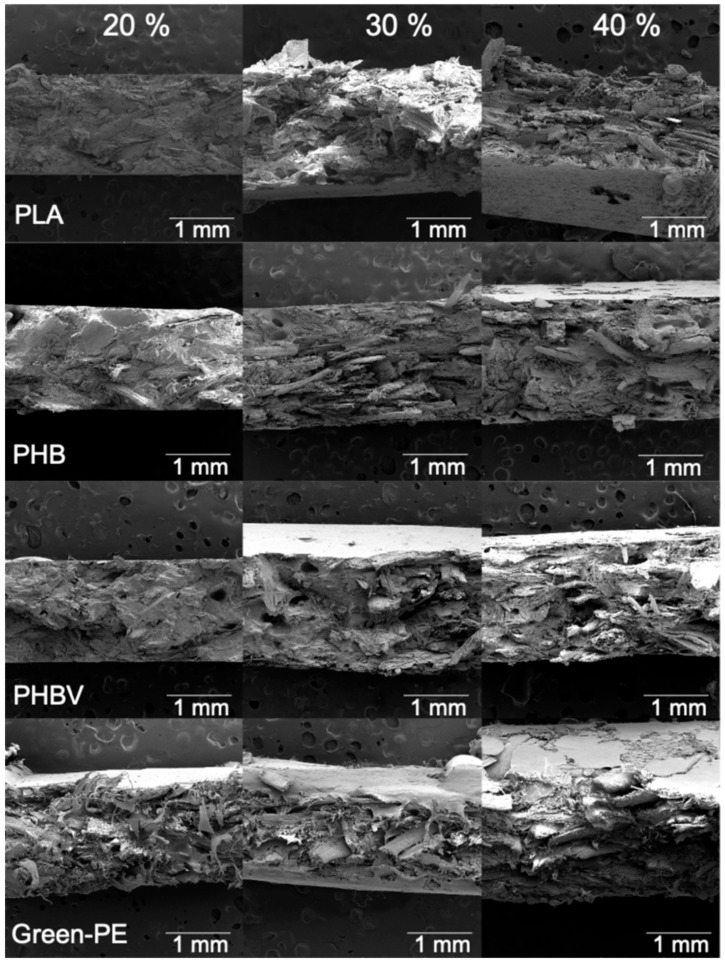
SEM micrographs of the biocomposites.

Figure 6 shows the biocomposites’ exposed fracture of the tensile specimens. The biocomposites with 20 wt.% of SCS showed fewer voids and fiber pull-outs than the materials with a higher SCS content. As expected, increasing the SCS content made the particle dispersion difficult and caused agglomerates, fiber pull-outs, and the formation of voids, affecting the stress transfer and, consequently, the tensile and flexural strengths.

**Figure 7:**
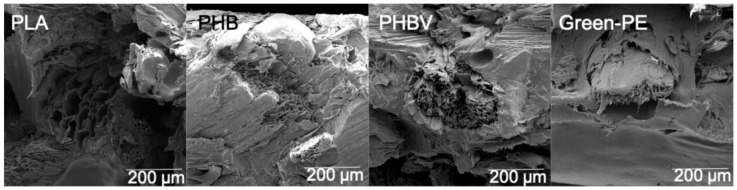
SEM micrographs of biocomposites at higher magnifications.

Figure 7 shows higher magnification micrographs of the biocomposites, aiming to observe the fiber–matrix interface. PLA, PHB, and PHBV morphologies showed good wettability of the SCS, which could be associated with a better interaction of SCS with these partially polar biopolymers [62,63]. This better compatibility explains the higher tensile and flexural strength values achieved with these matrices. On the other hand, Green-PE biocomposites showed a clear interfacial gap between the SCS and the matrix, indicating a poor adhesion between both components. These results confirmed that if a higher tensile and flexural strength are required for a specific application, it will be necessary to include a coupling agent or a surface treatment of the SCS.

### 3.3. Water Absorption

**Figure 8:**
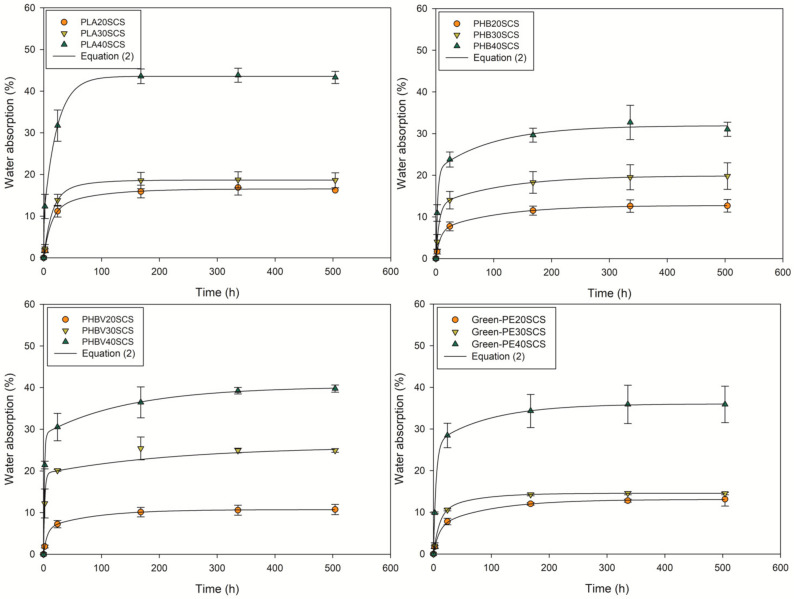
Water absorption curves of the biocomposites fitted to the hindered diffusion model.

The water absorption results are shown in Figure 8. The SCS significantly increased the water uptake of all biopolymers, having drastic increases at a 40 wt.% fiber content, especially in PLA and PHBV biocomposites, which reach a water uptake of 40%. The hydrophilic nature of SCS and its incompatibility with the matrix caused void formations through which water transport is made easier, making these materials very susceptible to water absorption [64]. Costa et al. [9] characterized the sugarcane straw, reporting water uptake values of 70%, so it was expected that biocomposites with 40 wt.% showed high water absorption values.

Figure 8 shows how the water absorption rate is slower for PLA and Green-PE, indicating that these materials are more resistant to water diffusion at short periods (lower than 24 h); such information agrees with the diffusion coefficients presented in Table 3. Lower water absorption values were obtained for Green-PE (10% for 20 and 30 wt.% of SCS) due to this high hydrophobicity of the matrix. In PHB and PHBV, the water absorption linearly increases with the fiber content, indicating that the humectation of the SCS with the polymer during compression molding is more homogeneous than with PLA and Green-PE composites, where drastic changes occur at 40 wt.% of SCS. These materials’ susceptibility to humidity is an important characteristic that must be considered for possible applications, especially for the biodegradable nature of some of these biopolymers.

## 4. Conclusions

This study aimed to explore the feasibility of SCS as a reinforcement/filler of different biopolymer matrices as an alternative method of reducing the final product cost. The obtained results were promising, as they showed the possibility of using this agro-industrial waste to prepare different biocomposites by a simple dry blending technique followed by compression molding, obtaining sustainable biocomposites with acceptable properties without substantially increasing processing costs. The characterization of the mechanical properties showed the following: the impact strength of PLA, PHB, and PHBV was not affected by the SCS (even at 40 wt.%), whereas, in Green-PE, the impact strength was slightly affected, but still retained competitive values. The tensile and flexural modulus were enhanced with 20 wt.% of SCS. However, when the concentration of SCS increases, the materials suffer a slight loss of these properties. The tensile and flexural strength were affected by all SCS contents due to the lack of compatibility. It is important to highlight that the obtained values were competitive against other conventional polymers (non-biodegradable and non-bio-based) despite the loss in some mechanical properties. The water absorption results showed that these materials have a high susceptibility to water, limiting their possible applications in highly humid environments. The results of this research attempt to offer an option to use the wasted SCS for possible commercial products without needing complex equipment or chemical processes.

## Figures and Tables

**Table 1:** Physical properties of biopolymers.

Biopolymer	Melt Flow Index (g/10 min)	Melting Temperature (°C)	Density (g/cm^3^)
PLA	35 *	155–170	1.24
PHB	8–15 *	175–180	1.25
PHBV	8–15 *	170–176	1.25
Green-PE	4.3 *	129	0.94

* 190 °C, 2.16 kg.

**Table 2:** Impact strength of different composite materials using the studied matrices.

Matrix	Blend Components	Impact Strength	Ref.
PLA	Sugarcane straw	27 J/m (13.5 kJ/m^2^)	This study
	Bamboo	9 kJ/m^2^	[34]
	Silicone/silane/polyol	30 J/m	[35]
	Kenaf	14 J/m	[30]
	Lyocell	52 J/m	
	Nanoclay	35 J/m	[36]
PHB	Sugarcane straw	23 J/m (11.5 kJ/m^2^)	This study
	Flax	65 J/m	[29]
	Kenaf	10 J/m	[30]
	Lyocell	70 J/m	
	Kenaf/oil palm empty fruit bunches	26 J/m	[37]
PHBV	Sugarcane straw	23 J/m (13.0 kJ/m^2^)	This study
	Wood	30 J/m	[38]
	Olive husk flour	0.45 kJ/m^2^	[39]
	Nanocellulose	4 kJ/m^2^	[40]
	Walnut shell	3 kJ/m^2^	
	Eggshell	5 kJ/m^2^	
Green-PE	Sugarcane straw	23 J/m (13.0 kJ/m^2^)	This study
	Wood	5.7 kJ/m^2^	[41]
	Basalt	10.5 kJ/m^2^	
	Flax	6 kJ/m^2^	
	Walnut shell	5.5 kJ/m^2^	
	Curaua	90 J/m	[42]
	Vermiculite clays	22 J/m	[43]

**Table 3:** Maximum water uptake of the materials and biocomposites diffusion coefficients calculated with the hindered diffusion model.

Sample	*M_∞_* (%)	*D* (10^9^ m^2^/s)
PLA	2.4	-
PLA20SCS	16.5	2.88
PLA30SCS	18.7	2.67
PLA40SCS	43.6	67.19
PHB	0.6	-
PHB20SCS	12.8	4.77
PHB30SCS	19.9	6.39
PHB40SCS	31.9	12.30
PHBV	0.4	-
PHBV20SCS	10.7	5.87
PHBV30SCS	25.9	17.45
PHBV40SCS	40.3	23.79
Green-PE	0.2	-
Green-PE20SCS	13.1	3.88
Green-PE30SCS	14.6	3.62
Green-PE40SCS	36.0	8.12

## Data Availability

The data presented in this study are available on request from the corresponding author.
